# Editorial: New evidence on the psychological impacts and consequences of COVID-19 on mental workload healthcare workers in diverse regions in the world

**DOI:** 10.3389/fpsyt.2023.1226793

**Published:** 2023-06-13

**Authors:** Stephen X. Zhang, Krystyna Kowalczuk

**Affiliations:** ^1^Adelaide Business School, University of Adelaide, Adelaide, SA, Australia; ^2^Department of Integrated Medical Care, Medical University of Bialystok, Białystok, Poland

**Keywords:** COVID-19, new normal, recovery, mental health, anxiety, distress, depression, PTSD

## Introduction

After 3 years, the COVID-19 pandemic has become the “new normal” around the world, and it is time to revisit the evidence and update the literature on the mental health status of various populations around the world. It's now crucial to reassess the empirical data and revitalize the existing body of literature concerning the mental health conditions of diverse populations worldwide. Updated insights offer an opportunity to update and compare these new findings with previous meta-analyses predominantly focused on the mental health status during the early stages of the pandemic (1–6). Such comparisons allow us to see whether mental health conditions continue to be as bad or have improved for various professions in various locations.

The papers published in this Special Issue has provided some new evidence, update and enrich the existing literature of meta-analytical evidence (1–11) as COVID-19 evolves. We especially request, whenever possible, it is necessary for the studies to follow standard research and reporting procedures such as PRISMA 2020 statement (12) and use common and validated measurement as in past meta-analyses on similar topics (13) to make comparisons effective and accumulate meaningful evidence. The published empirical papers covers different populations (for instance, employees, self-employed, unemployed, healthcare workers, nurses, and managers) from various geographical regions. The papers study certain populations and from certain regions but also more importantly compare and discuss the findings in relation to other related populations and other regions around the world (1–14) so that the discussion is not limited just to the author's region, thus embedding the study in the overall COVID-19 mental health literature.

## Summary of papers

Below, we provide a summary of the key findings of the papers in this special issue to provide readers with an overview of the research on this important topic.

Qin et al. conducted a study in China and found that burnout among healthcare professionals increased significantly during the COVID-19 pandemic, with higher levels observed among frontline workers. Socio-demographic factors such as age, work experience, and educational level were associated with burnout.

Makowicz et al. investigated the impact of the pandemic on job satisfaction among nurses in Poland, Germany, Italy, UK, and Sweden. The study found that the pandemic had a significant negative impact on job satisfaction among nurses in all five countries, with variations across the countries.

Jamebozorgi et al. conducted a study in the Middle East and found a high prevalence of depression, anxiety, and stress among healthcare workers across seven countries in the region. There were also significant differences in mental health outcomes among different countries.

Safwan et al. conducted a study in Lebanon and found a high prevalence of depression, anxiety, and stress among Lebanese pharmacists during the COVID-19 pandemic. The study also found associations between mental health and factors such as gender, age, marital status, and income.

Wang et al. explored the use of digital therapeutics for the treatment of mental health disorders. They discussed the potential benefits of digital therapeutics, provided examples of successful interventions, and highlighted challenges and future research directions.

Cayo-Rojas et al. discussed the impact of COVID-19 on the mental health of healthcare workers. They highlighted unique stressors faced by healthcare workers, such as fear of contracting the virus and ethical dilemmas, and emphasized the importance of support and resources for healthcare workers.

Osório et al. conducted a longitudinal study on the mental health and professional overload of health workers in Brazil during the first wave of the COVID-19 pandemic. They found high levels of psychological distress, burnout, and fatigue among health workers, associated with factors such as longer working hours, exposure to COVID-19 patients, and lack of workplace support.

Jaguga et al. focused on transcranial magnetic stimulation (TMS) as a treatment for depression, discussing mechanisms and evidence for its effectiveness. They highlighted the need for further research on optimal dosing and treatment protocols.

Chen et al. examined cognitive-behavioral therapy (CBT) as a treatment for anxiety disorders, discussing its theoretical basis and evidence for effectiveness. They concluded that CBT is a highly effective first-line treatment for anxiety disorders.

Ebrahimi Rigi et al. explored the bidirectional relationship between sleep and mental health, emphasizing the importance of addressing sleep problems in mental health treatment. Peng et al. investigated the relationship between night shifts, insomnia, anxiety, and depression among Chinese nurses during the COVID-19 pandemic, finding direct effects and associations between these factors.

Tong et al. highlighted the potential negative impact of excessive social media use on mental health during the COVID-19 pandemic. They suggested strategies to mitigate these effects, such as setting limits on social media use and engaging in offline activities. Hernández-Fernández and Meneses-Falcón discussed the use of social media to promote public health, highlighting benefits and drawbacks and emphasizing careful planning and evaluation.

Renzi et al. conducted a cross-sectional study on the mental health of Italian nurses during the second wave of the COVID-19 pandemic, finding high levels of anxiety, depression, and post-traumatic stress disorder (PTSD) symptoms. They identified female nurses and those with a history of mental health problems as higher-risk groups.

Ayalew et al. presented a cross-sectional study on the prevalence of PTSD symptoms among healthcare workers in southern Ethiopia following the COVID-19 pandemic. They found high levels of PTSD symptoms and identified female healthcare workers and those with a history of mental health problems as higher-risk groups.

Chung et al. investigated the influence of intolerance of uncertainty and viral anxiety on adherence to physical distancing among healthcare workers during the COVID-19 pandemic. They found a negative association between intolerance of uncertainty and physical distancing adherence, partially mediated by viral anxiety.

Ahn et al. validated the Grief Support in Healthcare Scale (GSHS) among frontline nursing professionals in Korea and found associations between grief support, emotional exhaustion, and job satisfaction.

Yi et al. conducted a cross-sectional study on the impact of the COVID-19 pandemic on non-frontline pediatric nurses in China, finding high levels of acute stress reaction, depression, anxiety, and stress. They identified job withdrawal behaviors associated with mental health issues.

Sun et al. conducted a cross-sectional study on the psychological status of medical staff involved in nucleic acid collection during the COVID-19 epidemic in China, finding high levels of anxiety, depression, and stress associated with lack of social support, exposure to COVID-19 patients, and concerns about personal safety.

## Implications and future research directions

In conclusion, the collective findings of these studies underscore the profound impact of the COVID-19 pandemic on the mental health and wellbeing of diverse populations, across COVID waves and various countries and regions—see a definition of COVID-19 waves here (15). These findings serve as a clarion call that we proactively monitor the wellbeing and mental health of individuals and communities during and beyond the COVID-19 pandemic, particularly as countries have been closed and then reopened (16). This continuous monitoring will enable timely interventions and support systems to safeguard mental health and wellbeing in the face of ongoing challenges. Moreover, the studies emphasize the urgent need for further research to deepen our understanding of the multifaceted factors contributing to mental health outcomes among diverse populations and across different countries. By gaining comprehensive insights into these factors, we can inform evidence-based interventions and update meta-analyses, ensuring that future epidemics are met with targeted strategies that prioritize mental health of the more vulnerable populations.

## Author contributions

All authors listed have made a substantial, direct, and intellectual contribution to the work and approved it for publication.
